# Towards markerless surgical tool and hand pose estimation

**DOI:** 10.1007/s11548-021-02369-2

**Published:** 2021-04-21

**Authors:** Jonas Hein, Matthias Seibold, Federica Bogo, Mazda Farshad, Marc Pollefeys, Philipp Fürnstahl, Nassir Navab

**Affiliations:** 1 Research in Orthopedic Computer Science, University Hospital Balgrist, University of Zurich, Balgrist CAMPUS, Zurich, Switzerland; 2 Computer Vision and Geometry Group, ETH Zurich, Zurich, Switzerland; 3 Computer Aided Medical Procedures, Technical University Munich, Garching, Germany; 4 Mixed Reality & AI Zurich Lab, Microsoft, Zurich, Switzerland; 5 Balgrist University Hospital, University of Zurich, Zurich, Switzerland

**Keywords:** Object pose, Hand pose, Single-shot pose estimation, Synthetic data generation, Deep learning

## Abstract

**Purpose:**

Tracking of tools and surgical activity is becoming more and more important in the context of computer assisted surgery. In this work, we present a data generation framework, dataset and baseline methods to facilitate further research in the direction of markerless hand and instrument pose estimation in realistic surgical scenarios.

**Methods:**

We developed a rendering pipeline to create inexpensive and realistic synthetic data for model pretraining. Subsequently, we propose a pipeline to capture and label real data with hand and object pose ground truth in an experimental setup to gather high-quality real data. We furthermore present three state-of-the-art RGB-based pose estimation baselines.

**Results:**

We evaluate three baseline models on the proposed datasets. The best performing baseline achieves an average tool 3D vertex error of 16.7 mm on synthetic data as well as 13.8 mm on real data which is comparable to the state-of-the art in RGB-based hand/object pose estimation.

**Conclusion:**

To the best of our knowledge, we propose the first synthetic and real data generation pipelines to generate hand and object pose labels for open surgery. We present three baseline models for RGB based object and object/hand pose estimation based on RGB frames. Our realistic synthetic data generation pipeline may contribute to overcome the data bottleneck in the surgical domain and can easily be transferred to other medical applications.

**Supplementary Information:**

The online version supplementary material available at 10.1007/s11548-021-02369-2.

## Introduction

Visual 3D pose recognition of surgical tools [1], the patient anatomy [3], but also of the surgical staff [25] in video data is becoming increasingly important in clinical research. However, regulations and the risk of patient compromise make it challenging to collect sufficient amounts of training data to develop robust and generalizable methods [8]. As a consequence, there are no publicly available clinical datasets yet. Monocular RGB video is still the most common optical system used in today’s operating rooms and is employed for surgical education, performance enhancement, and error analysis [32].

Instrument pose estimation is an essential part of computer aided surgery and is deployed in state-of-the-art surgical navigation systems [7, 12], as well as augmented reality systems [20, 24, 27], through optical tracking to localize a surgical instrument in the 3D space of the operating theatre. The spatial localization of the tool combined with a co-registration of a preoperative plan or intra-operative medical imaging to the patient anatomy enables surgical guidance to improve the outcome of the intervention and reduce the radiation exposure for both the patient and the surgical staff [22, 39]. As the tool is always partly occluded by the hand, when in use, taking hand tracking into account could be beneficial to estimate the tool pose. In addition, more than $70\%$ of intraoperative complications (iatrogenic injuries) are related to the surgical treatment itself [9, 10]. 3D pose estimation of surgical tools in regard to the patient anatomy enables the prevention of surgical errors and potentially reduces the risk for iatrogenic injuries by detecting proximity to risk structures, e.g., during drill task execution. Furthermore, joint hand and tool tracking opens up possibilities for tracking surgical activity which can be used for workflow recognition [25] or skill assessment and training of surgeons [11].

In this work, we introduce a novel clinical dataset, consisting of a synthetic and a real subset of RGB frames and corresponding hand and object pose labels to enable the development of tool pose and hand pose estimation solutions for the medical domain. Since there are no publicly available datasets for this novel domain, we propose a pipeline to generate inexpensive but realistic synthetic data. Pretraining using synthetic data has been shown to achieve good results when the availability of annotated real data is limited [36]. Furthermore, we present a semi-automatic labeling method which allowed us to create a second dataset based on real recordings captured in a mock operating room.

The target object used in our dataset is a surgical drill, as bone drilling is conducted in about 95% of orthopedic interventions [2] and is a highly relevant clinical procedure. We propose three baseline models for object and combined object–hand pose estimation based on RGB frames for seamless integration into current surgical workflows. The presented work introduces the problem and facilitates further research toward markerless tool and hand pose estimation in a surgical scenario.

## Related work

In this section, we present the related work in the context of 3D pose estimation of tools and hands, covering both separate and combined approaches, as well as previous work in the field of synthetic pretraining in supervised deep learning.

Object pose estimation in RGB images is nowadays mostly accomplished by using convolutional neural networks (CNNs) and has been shown to yield promising results [6, 18, 38]. Instead of directly regressing the object pose, models are often trained to regress 2D keypoints. The keypoints are used to recover the 6D object pose by applying the perspective-n-point (PnP) algorithm. One of the current state-of-the-art object tracking models, PVNet [26], utilizes this technique and performs well even under occlusions.

Several approaches have been proposed to estimate the hand pose and hand configuration based on single-frame RGB inputs [14, 34]. A hand model, e.g., the parametric MANO model [29], enforces the biomechanical plausibility of the estimated hand configuration and is commonly used in many hand tracking approaches. The MANO hand model deforms a 3D hand mesh template according to a set of pose and shape parameters. The pose and shape parameters correspond to the principle components of the pose and shape space, respectively, which were computed from a dataset of high-resolution hand scans.

Joint tracking of a hand and object in interaction is still a very recent field of research. Compared to object-only or hand-only pose estimation, the close proximity of hand and object makes the task particularly challenging due to mutual occlusions. Tekin et al. [35] proposed a model for hand–object pose estimation as well as action recognition. Hasson et al. estimate the pose of hand and object simultaneously and reconstruct a mesh representation for both [15]. In their follow-up work, they proposed a joint hand–object pose estimation model, which directly regresses the object pose as well as the MANO pose and shape parameters with sparse supervision [14]. None of the approaches mentioned in this section have been applied in the medical domain, which is also due to the lack of publicly available datasets in this domain. Compared to the previous work, the surgical scenario introduces additional challenges, such as lighting conditions and strong hand–object occlusions when holding a surgical instrument, e.g., a medical drill.

In scenarios where it is practically unfeasible or very expensive to collect large amounts of labeled real data, synthetic pretraining approaches have been shown to yield promising results for supervised learning-based pose estimation [15, 33]. The performance of the model can be increased when the network is refined with a small amount of real data [36]. Also in the medical domain, synthetic pretraining has been shown to have beneficial results on the model accuracy [31].

## Methodology

In the following paragraphs, we present our synthetic data generation pipeline, the setup and methods used for capturing a real-world dataset in a mock operating room, as well as three baseline models for RGB-based tool and hand pose estimation. However, the current state-of-the-art in computer-assisted surgery lacks rendering pipelines capable of generating realistic images that can be successfully used for pretraining. All checkpoints, code and datasets have been made public for further research and reproducibility^1^.

### Synthetic data generation

There are several requirements for the synthetic data generation. In order to keep the domain gap as small as possible, the synthetically generated data have to follow the underlying statistics of real data as closely as possible. For image data, this requirement implies that the generated images have to look realistic and visualize a variety that is similar to real-world situations.

**Fig. 1:**
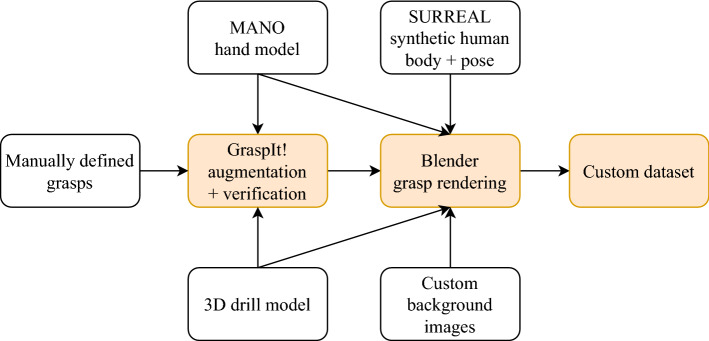
Schematic overview of the synthetic data generation pipeline

Our synthetic data generation pipeline is based on the implementation by Hasson et al. [15] but adapted to our specific scenario in the surgical domain. First, a set of 7 bio-mechanically plausible, tool-specific random grasps is generated using the MANO model [29] and the *GraspIt!* Simulator [23]. An accurate 3D model of a Colibri II battery powered drill (DePuy Synthes, Raynham, MA, USA) was reverse-engineered from a CT scan by manual segmentation in *Slicer3D* and texturing in *Blender* (Stichting Blender Foundation, Amsterdam, Netherlands). Similar to Hasson et al. [15], we utilize the SMPL+H [29] model, which is a combination of the SMPL [21] human body model and the MANO hand model. The hand pose parameters of the SMPL+H model are set to the generated grasp values, while the remaining body pose parameters as well as body textures are randomly sampled from the SURREAL [37] dataset. Using the SMPL+H model, we can increase the realism of the rendered scene by connecting the hand to a body and by placing the camera at the approximate position of the head. The hand texture is set to a constant blue color that resembles surgical gloves. In addition, the scene lighting is adjusted for the extreme lighting conditions in an operating room, with bright and focused spotlights and a comparably low general illumination without natural sunlight. We render the scene in *Blender* using the physically based *Cycles* renderer. Custom background images are added from a real spine surgery video that was recorded with a head-mounted camera. The outline of the synthetic data generation pipeline is illustrated in Fig. 1.

Additionally, we assume that the rough location of the hand or object has already been estimated, e.g., via a hand or object detection model [28]. Thus, we generate patches that are roughly centered on the hand. We constrain the viewpoint to an egocentric perspective and randomize the exact position and orientation slightly by adding uniform noise from the intervals $[-0.1, 0.1]$ and $[-0.02, 0.02]$ to the head and hand position, respectively, before placing the virtual camera on the augmented head position and pointing it toward the augmented hand position. The distance between camera and drill is uniformly sampled from 30 cm to 50 cm.

**Fig. 2:**
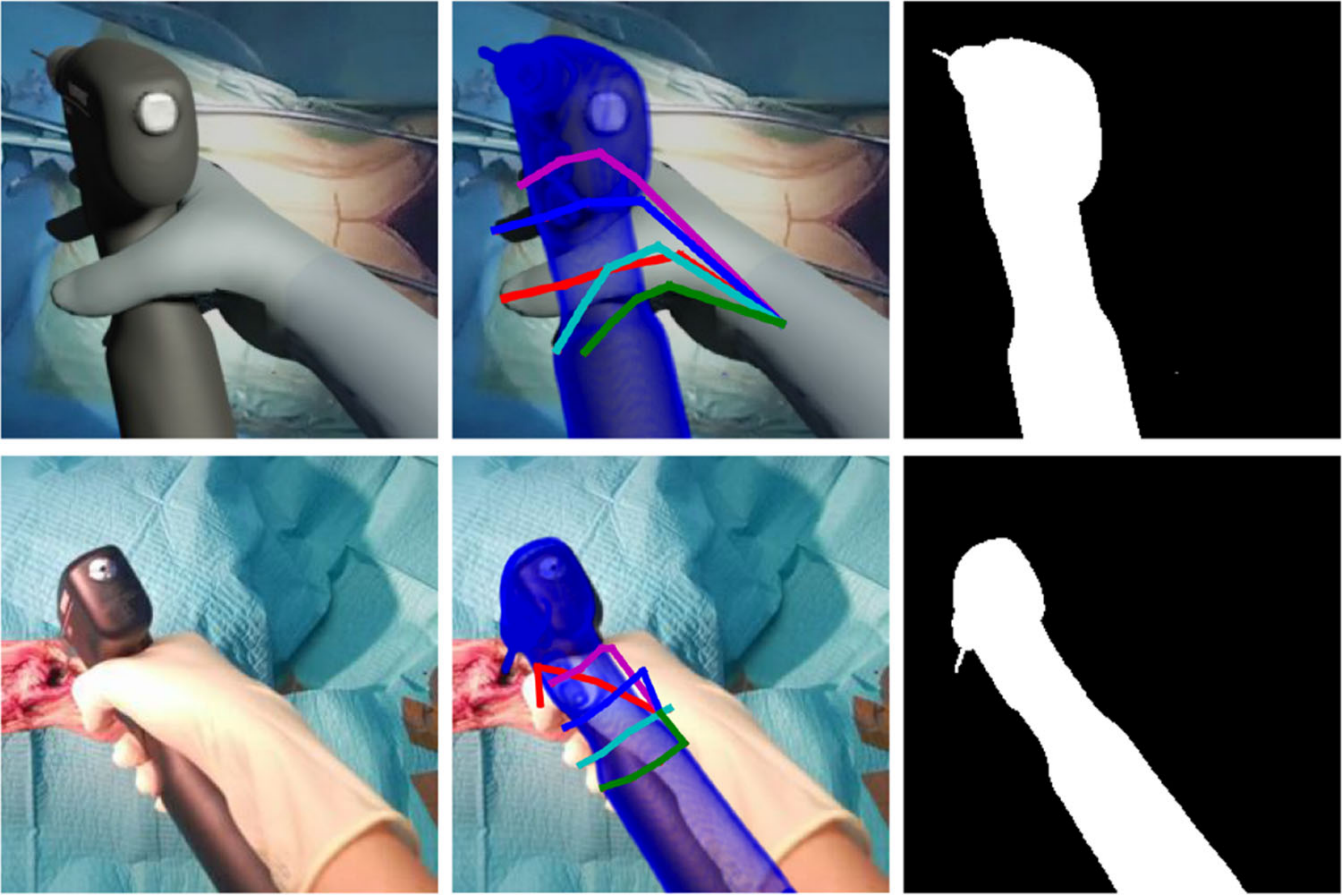
One example of a RGB image (left), the respective ground truth and segmentation mask (right), taken from the synthetic dataset (top row) and the real dataset (bottom row)

Besides the RGB image, a segmentation mask is rendered for each sample (Fig. 2). To ensure that the hand and object are at least partly visible, we evaluate the hand and object segmentation masks and discard all renderings where less than 100 pixels belong to hand or object, respectively. Additionally, we exclude invalid configurations (e.g., when the camera is positioned inside the body model) by filtering out renderings where less than 40% of the object is visible. We manually define 7 grasps templates in the *GraspIt!* Simulator to account for slight differences in the user’s grasp of the drill, such as the number of fingers placed on the buttons. We generate a set of 210 augmented grasps by repeatedly sampling a random grasp template and adding Gaussian noise to the hand pose ($\sigma = 0.01$) and shape ($\sigma = 0.05$) parameters in order to increase the diversity of the dataset. The augmented grasps are verified to be physically and biomechanically plausible.

We render a total of 10500 samples based on the augmented grasps. The rendered frames have a resolution of $256 \times 256$ pixels.

### Real data generation

Even though synthetic data are inexpensive to generate in large amounts, it is only an approximation of the true data domain. Therefore, using synthetic data for pretraining opens up a domain gap between the synthetic and real data domain. We generated a real dataset in a mock operating room and refine the synthetically pretrained models, and evaluate them on a real test set.

A human cadaveric specimen with an open incision was placed on the operating table and covered in surgical drapes to make the scenario as realistic as possible. Two users were asked to perform handling of the drill in the surgical workspace. The scene was captured with a setup of two stereo-calibrated and hardware-synchronized Azure Kinect DK cameras (Microsoft Corporation, Redmond, WA, USA). To simplify the generation of ground truth annotations as well as to increase their accuracy, the cameras captured the scene from orthogonal viewpoints. We acquired RGB and depth frames and reconstruct the colorized overlapping point clouds for ground truth labeling while handling a real Colibri II drill. We choose this marker-less tracking approach over marker-based approaches to recover the ground truth hand and tool poses, since any markers attached to the tool or hand would be visible in the captured images and can introduce a bias for learning based methods [13].

The ground truth object labels are generated as follows. For each recording, we ensure that the drill is only picked up once at the beginning and the grasp is not altered during recording the sequence.

The 3D vertices of the tool model ${\mathbb {V}}_{\text {tool}} \in {\mathbb {R}}^{\text {N} \times 3}$ are registered to an initial point cloud frame ${\mathbb {P}}^0$. Then, we manually select all hand points ${\mathbb {P}}^0_{\text {hand}} \subset {\mathbb {P}}^0$ from the point cloud and merge them with the tool vertices to create a joint hand-tool point cloud ${\mathbb {P}}_{\text {joint}} = {\mathbb {V}}_{\text {tool}} \cup {\mathbb {P}}^0_{\text {hand}}$. Due to the fact that the tool surface in the point cloud is often incomplete, which is likely due to the drill’s matt plastic material, taking the hand point cloud into account by registering the joint hand-tool model ${\mathbb {P}}_{\text {joint}}$ greatly improves the stability of the ICP-based pose registration process. Next, the combined model ${\mathbb {P}}_{\text {joint}}$ is registered to the remaining point cloud frames ${\mathbb {P}}^1, ... {\mathbb {P}}^T$ of the recording, using the trimmed ICP variant by Chetverikov et al. [5]. To recover the tool pose $H^t_{\text {tool}} \in {\mathbb {R}}^{4 \times 4}$ from the point cloud ${\mathbb {P}}^t$, we initialize the ICP algorithm with the previous frame’s pose $H^{t-1}_{\text {tool}}$. Additionally, we re-initialized registration in case ICP diverges from the true tool pose and rerun the registration from that frame. Last, we manually sight the results and discard frames with inaccurate labels.

The ground truth hand labels are recovered based on the joint hand-tool model. We define a set of 16 vertices on the MANO hand mesh and manually label corresponding points on the hand-tool model ${\mathbb {P}}_{\text {joint}}$. Then, we recover the hand pose $H^0_{\text {hand}} \in {\mathbb {R}}^{4 \times 4}$ as well as the PCA pose parameters $\theta$ of the MANO model by minimizing the pairwise distance between the labeled points and vertices. We do not optimize the hand shape parameters, but assume the average hand shape ($\beta = 0$). To ensure biomechanical plausibility, we $\ell _2$-regularize the pose parameters. Last, the per-frame hand labels (in the camera coordinate frame) are recovered via $H^t_{\text {hand}} = H^t_{\text {tool}} H^0_{\text {hand}}$.

Since our model takes image patches instead of full-HD images as an input, the main camera’s RGB image is cropped around the 2D center of the drill. We define the 2D center of the drill as the center of its 2D bounding box. The true 2D center is augmented by randomly shifting it up to 64 pixels in a random direction. Prior to cropping the image, we compensate for any difference in the focal lengths of the Kinect and the simulated camera by scaling the image accordingly. We discard all patches which show less than $40 \%$ of the tool’s projected 2D vertices, effectively removing cases of extreme truncation. Last, we reduce the sampling rate to 5 frames per second to increase the difference of consecutive frames.

Our final dataset consists of 3746 frames which are extracted from a total of 11 individual recordings. To increase the diversity of the recordings, the drill is operated by two different users wearing one of two differently colored pairs of rubber gloves. The cropped image patches have a size of $256 \times 256$ pixels. Each frame is annotated with the 6D tool pose, as well as the 3D hand joints in camera coordinates.

### Baseline models

#### PVNet

We choose PVNet [26] as the first baseline since it is a state-of-the-art model for object-only pose estimation on single-shot RGB images. Furthermore, the model has been shown to be robust against occlusions, which is particularly important in our use-case due to the expected occlusions caused by the surgeon’s hand.

Instead of directly regressing translation and rotation parameters of the 6D object pose, PVNet indirectly estimates the object pose via a set of $K=8+1$ 2D keypoints, which correspond to predefined 3D locations on the object’s surface as well as the object’s bounding box center. The 3D locations are sampled using farthest point sampling in order to increase the stability of the PnP algorithm which is used to recover the 6D object pose. PVNet employs a U-Net [30] like model architecture that is used to estimate a 2D vector field for each keypoint, as well as a segmentation mask.

The predicted 2D keypoints are recovered from their vector field representation using a RANSAC-based voting scheme. Keypoint hypotheses are repeatedly computed by triangulating two random vectors from the vector field which belong to the same object instance according to the estimated segmentation mask. The quality of the triangulated keypoint hypothesis is estimated by counting the number of inliers in the vector field. Additionally, the mean and covariance of the generated keypoint hypotheses are computed, where each keypoint hypothesis is weighted by its inlier count.

The final 6D tool pose ${\hat{R}}, {\hat{t}}$ is recovered via an uncertainty-driven PnP approach that minimizes the Mahalanobis distance

1 $$\begin{aligned} {\hat{R}}, {\hat{t}} = \min _{R, t} \sum _{k=1}^K (\hat{{\mathbf {x}}}_k - \varvec{\mu }_k)^T \varSigma _k^{-1} (\hat{{\mathbf {x}}}_k - \varvec{\mu }_k), \end{aligned}$$

between the estimated keypoint distributions $(\varvec{\mu }_k, \varSigma _k)$ and the ground truth 2D keypoints $\hat{{\mathbf {x}}}_k$. The initial guesses for the rotation *R* and translation *t* are computed via EPnP [19] based on the four keypoints with the lowest uncertainty. Additional details on the RANSAC voting scheme and the uncertainty-driven PnP approach are provided in [26].

During training, we set the hyperparameters to the optimal values as reported in [26]. The PVNet model is trained with a batch size of 8, a learning rate of ${1\times 10^{-3}}$, and the ADAM optimizer with a momentum of 0.9. The learning rate is halved every 20 epochs.

#### HandObjectNet

We additionally evaluate the single-frame hand–object reconstruction network (HandObjectNet) by Hasson et al. [14] as a second baseline. In contrast to PVNet, HandObjectNet jointly estimates the poses for hand and object, which can potentially improve its accuracy if the model learns implicit grasp characteristics, such as the relative pose of the tool with respect to the hand. Such grasp characteristics enable the model to also indirectly estimate the tool pose, which becomes important when large parts of the tool are occluded by the hand.

HandObjectNet consists of a shared ResNet-18 [16] encoder and two decoders for the hand and object, respectively. The hand decoder uses two branches of fully connected layers with ReLU activations to estimate the hand pose and shape parameters. The first branch estimates 18 pose parameters $\theta$ and 10 shape parameters $\beta$ of the MANO model. The pose parameters consist of 15 principle component coefficients which define the hand configuration and 3 parameters that encode the global hand rotation in an axis-angle format.

The second branch regresses a 2D translation vector $\hat{{\mathbf {t}}}' \in {\mathbb {R}}^2$ of the hand in the image, and a focal-normalized depth offset

2 $$\begin{aligned} d_f = \dfrac{{\mathbf {t}}|_z - z_0}{f} \in {\mathbb {R}}, \end{aligned}$$

where ${\mathbf {t}}|_z$ is the depth component of the 3D translation between the hand and the camera, *f* is the (known) focal length, and $z_0$ is a depth offset set to 0.4 meters, as proposed in [14], in order to roughly normalize the depth estimates. To simplify the recovery of the 3D hand translation, we assume that the principle point of the camera is located in the image center. This assumption holds for both of our datasets. Then, the estimated 3D translation of the hand is

3 $$\begin{aligned} \hat{{\mathbf {t}}} = \begin{pmatrix} \dfrac{\hat{{\mathbf {t}}}'|_x \hat{{\mathbf {t}}}|_z}{f}&\dfrac{\hat{{\mathbf {t}}}'|_y \hat{{\mathbf {t}}}|_z}{f}&\hat{{\mathbf {t}}}|_z \end{pmatrix}^T \in {\mathbb {R}}^3, \hat{{\mathbf {t}}}|_z = f {\hat{d}}_f + z_0. \end{aligned}$$

During training, we set the hyperparameters to the same values as reported in [14]. We train HandObjectNet with a batch size of 8, a learning rate of ${5\times 10^{-5}}$, and the ADAM optimizer with a momentum of 0.9.

#### Combined model

We furthermore propose a third baseline which is a combination of HandObjectNet [14] and PVNet [26]. We motivate this combination with the robustness of this indirect pose estimation via keypoints, which can potentially further improve the accuracy of HandObjectNet. Instead of directly regressing the 3D object pose via fully connected layers, such as employed in HandObjectNet, we propose to adopt the pose estimation method by using vector field encoded keypoints, similar to the method introduced for PVNet [26]. Hereby, we replace the HandObjectNet’s object decoder branch with PVNet’s object decoder branch, including the RANSAC voting scheme and the uncertainty-driven PnP. We also adopt the skip connections between the layers of the encoder and the object decoder to keep the U-Net like architecture intact. A detailed visualization of the proposed model architecture can be found in the supplementary materials.

We train all models using the PyTorch framework. The brightness, contrast, hue and saturation of the training samples are randomly augmented to prevent overfitting. We further apply early stopping by evaluating the model on the validation set. During training, we optimize the combined model using the ADAM optimizer with a learning rate of ${5\times 10^{-5}}$, a momentum of 0.9 and a batch size of 64 which are empirically determined hyperparameters.

## Results and evaluation

**Table 1:** Comparison of the model accuracy on the synthetic test set.

$\downarrow$ Metric, Model $\rightarrow$	HandObjectNet [14]		PVNet [26]		Ours	
	Mean	SD	Mean	SD	Mean	SD
Tool ADD (mm)	**16**.**73**	16.97	20.59	52.14	32.51	72.72
Tool Proj2D (px)	**13**.**65**	15.65	15.59	250.91	16.84	202.44
Drill tip error (mm)	44.45	59.72	**31**.**10**	67.18	44.16	86.79
Drill bit direction error (deg)	**6**.**59**	10.18	7.11	21.78	8.64	22.77
2D keypoint error (px)	–	–	12.30	13.08	16.08	16.33
Hand ADD (mm)	**17**.**15**	10.58	–	–	19.07	11.68
Hand Proj2D (px)	**13**.**44**	7.75	–	–	15.15	8.94

All models were trained exclusively on synthetic data. We report the averaged mean and standard deviation of fivefold cross-validation. Bold values indicate best performance

**Table 2:** Results after refinement on real data. All models were trained on synthetic data and refined using real data. We report the averaged mean and standard deviation of fivefold cross-validation. Bold values indicate best performance

$\downarrow$ Metric, Model $\rightarrow$	HandObjectNet [14]		PVNet [26]		Ours	
	Mean	SD	Mean	SD	Mean	SD
Tool ADD (mm)	**13**.**78**	5.28	39.72	66.49	39.43	70.38
Tool Proj2D (px)	**10**.**36**	14.52	12.83	51.26	13.77	69.73
Drill tip error (mm)	**66**.**11**	26.91	72.80	105.66	72.91	116.70
Drill bit direction error (deg)	**8**.**71**	3.98	13.41	33.78	14.61	36.78
2D keypoint error (px)	–	–	11.77	10.16	12.13	16.68
Hand ADD (mm)	**9**.**78**	4.54	–	–	21.68	13.96
Hand Proj2D (px)	**6**.**14**	7.69	–	–	12.99	10.80

We evaluate all baselines after pretraining with synthetic data as well as after refinement on real data. We use fivefold cross-validation to measure the variance between different splits and ensure the statistical significance of the reported results. The synthetic and real datasets are split on the level of augmented grasps and recordings, respectively.

### Synthetic data

**Fig. 3:**
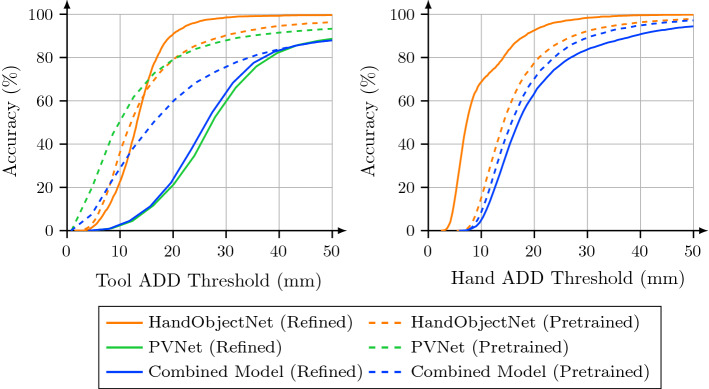
Accuracy–threshold curves of the tool and hand ADD metrics for all baseline models. Pretrained models are indicated with dashed lines and evaluated on synthetic data. Refined models are indicated with solid lines and evaluated on real data

We train and evaluate the three baseline models, PVNet [26], HandObjectNet [14] and the combined model on the proposed synthetic dataset. For evaluation, we use the ADD metric [17], which is the average 3D error between corresponding vertices of the tool mesh. We additionally evaluate the 2D projection error [4] of the tool vertices. The ADD and Proj2D metrics are evaluated on the tool vertices as well as the hand joints (Fig. 3). As an important measure for the drilling process, we report the position error of the drill tip and the angular error w.r.t. the direction of the drill bit.

The results reported in Table 1 show that for the pretraining with synthetic data, HandObjectNet outperforms the combined model and PVNet and achieves the lowest average error across all metrics with the exception of the drill tip error.

### Real data and fine tuning

We first evaluated the performance of the models which were trained exclusively on synthetic data. We observed that all models suffer from huge performance decreases due the synthetic-real domain gap and are therefore not suited for the application on real data without further refinement.

To reduce this domain gap, we refine the models on the real training set after pretraining them on the synthetic dataset. We observe that both PVNet and the combined model predict few samples with extraordinary large depth errors exceeding ${1\times 10^{12}}$ m due to incorrect keypoint estimates. To remove these outliers, we introduce a post-processing step that discards invalid predictions with a distance of more than ${1\times 10^{3}}$ m from the camera. For either model less than 10 samples or $0.2\%$ of the dataset are discarded.

We report the results of all baselines after refinement with real data in Table 2. HandObjectNet clearly outperforms the other two baselines on all metrics, although there is significant variance in the drill tip error. Compared to its accuracy on the synthetic dataset after pretraining, HandObjectNet achieves a higher accuracy on the real dataset after refinement. We attribute this performance increase to a generally lower variance of the real dataset. In contrast, PVNet and the combined model yield less consistent results, as they fail to reliably estimate the 2D keypoints, which introduces large errors during the pose recovery via PnP. Additional qualitative and quantitative results of all baselines can be found in the supplementary materials.

## Discussion

Since pretraining with synthetic data has been shown to be a beneficial approach, especially for scenarios in which real-world data collection is expensive, we propose a pipeline to inexpensively generate realistic synthetic RGB frames of instrument–hand interaction in surgical scenarios. We furthermore developed a standardized setup to capture and label real-world data with object and hand pose and generated a realistic dataset in a mock operating room. This combination of synthetic and real data generation paves the path for markerless object and hand pose estimation in surgery. To this end, we propose three baseline models, train them on the generated datasets and evaluate them using fivefold cross-validation.

The best performing baseline achieves an average 3D vertex error of 16.7 mm on synthetic data as well as 13.8 mm on real data. These results are in line with the results from the current state-of-the-art from computer vision applications, such as reported in [15, 26]. HandObjectNet yields consistent results and clearly outperforms the other baselines, while PVNet shows larger errors after refinement with real data which occurs due to high uncertainties in the keypoint estimation. The combined model shows very similar performance with the PVNet baseline. In contrast to PVNet and the combined model, HandObjectNet performs more robustly throughout the test sets, which is illustrated in qualitative examples in the supplementary materials.

Even though the synthetic data generation pipeline proposed in this work generates realistic samples, there is an observable domain gap which manifests itself in decreasing performance from synthetic to real data for all three baselines. This is caused by different underlying distributions of the synthetic and real dataset, for example, visual discrepancies such as illumination, contrast or color. We used background frames from a recording of a spinal surgery recording to improve the realism of the synthetic samples; however, the mock operating room, in which the real dataset was recorded, provided different lighting conditions.

Adding further modalities has significant potential to improve the model’s performances, e.g., by including depth sensors which offer additional depth information compared to monocular video. However, there are several challenges for the use of RGB-D data in real-world surgery, such as the short distance to the observed target and the exposure under challenging lighting conditions [15], which are present in the operating room. An alternative to RGB-D cameras is stereo RGB cameras or multi-view camera systems, which provide additional information through a second view instead of infrared-based depth measurements, which are often noisy. However, the bulkiness of wide-baseline stereo RGB cameras can introduce logistical problems to the OR, while multi-camera systems generally have to be carefully calibrated and must not be moved afterward, which strongly limits the placement of these cameras in close proximity to the operating table. On the other hand, motivations for utilizing RGB video data are manifold, such as the independence from the deployed camera technology, the resulting possibility for the analysis of retrospective surgical video data, the availability of medical device certification for video cameras, or the option to use cheap single-use off-the-shelf cameras.

To improve the accuracy of our method and therefore the applicability for surgical scenarios, in future work, we want to investigate RGB-D-based markerless object and tool pose estimation. Therefore, the proposed synthetic and real data generation pipelines have to be extended to include depth data. Even though this approach requires specialized hardware in the operating room, there is potential to increase the performance of the pose estimation algorithm. We furthermore want to increase the variety and size of the datasets, e.g., by changing the lighting conditions.

Currently, the synthetic data generation pipeline, as well as the proposed baseline methods are designed for single-shot pose estimation and do not incorporate sequence data. To overcome this current limitation, we want to extend the data generation as well as the pose estimation methods to work with time-varying sequence data, which could potentially increase the performance. Furthermore, the synthetic pipeline can be adapted easily for other medical scenarios by replacing the 3D model and background images. Another limitation of the presented work is that all data were acquired in a known coordinate frame. For the use in real-world surgery, head-mounted cameras require additional tracking solutions which introduce additional errors that should be subject to further research.

## Conclusion

In this work, we present two pipelines for synthetic and real training data generation, a novel dataset and three baseline models for joint hand and tool pose estimation based on RGB image data targeted for surgery. Synthetic pretraining is a promising approach, especially for the medical domain, where it is often expensive to generate a sufficient amount of real training data. Pose estimation in RGB frames offers the possibility of seamless integration into the current workflows of the operating room, but misses depth information in contrast to RGB-D cameras. The obtained results of the proposed baseline models are in line with the current state of the art, but not yet suited for surgical tracking applications.

The datasets and baselines proposed in this work pave the path for a single approach for tool tracking, surgical error prevention, as well as activity and workflow recognition by simultaneously detecting the tool pose and the surgeon’s hand pose and shape. Future work includes adding depth information, investigating sequence data and increasing the variety and size of the datasets.

## Supplementary Information

Below is the link to the electronic supplementary material.

Supplementary material 1 (pdf 5473 KB)

## Footnotes

1 http://medicalaugmentedreality.org/handobject.html
