# Supplementary material for: Towards markerless surgical tool and hand pose estimation
Source: Int J Comput Assist Radiol Surg. 2021 Apr 21;16(5):799–808. doi: 10.1007/s11548-021-02369-2 (PMC8134312; doi:10.1007/s11548-021-02369-2)
Supplement: Supplementary file 1 — Supplementary material 1 (pdf 5473 KB) [file 11548_2021_2369_MOESM1_ESM.pdf]

Noname manuscript No.
(will be inserted by the editor)

Towards markerless surgical tool and hand pose estimation - Supplementary Materials

***Jonas Hein · *Matthias Seibold**
Federica Bogo · Mazda Farshad
Marc Pollefeys · †Philipp Fürnstahl
†Nassir Navab

the date of receipt and acceptance should be inserted later

1 Introduction

To complement the main manuscript, we provide further explanations, illustrations, and details about the architecture of the combined model and runtime of all networks in this supplementary document. Furthermore, the results section contains additional evaluations of the three baseline models trained on the proposed synthetic and real datasets.

2 Baseline models

In the following paragraphs we provide additional information about the architecture of the combined model, as well as implementation and runtime details of the proposed baselines.

2.1 Combined Model

Figure 1 illustrates the combined model architecture, as described in section 3.3.3 in the main manuscript. This model is a combination of the other two state-of-the-art baselines, *PVNet* and *HandObjectNet*. It consists of a shared ResNet-18 encoder and two decoders for the hand and tool pose, respectively.

The encoder together with the PVNet object decoder resemble a U-Net-like structure [4], which is explained in detail in the following paragraph. The object decoder is a series of blocks consisting of a bilinear upsampling layer and a convolution with batch normalization and a leaky ReLu activation function. Each

✉ E-mail: heinj@student.ethz.ch, matthias.seibold@tum.de

* Jonas Hein and Matthias Seibold contributed equally to this work and are listed as co-first authors in alphabetical order.
† Philipp Fürnstahl and Nassir Navab are listed as co-last authors in alphabetical order.

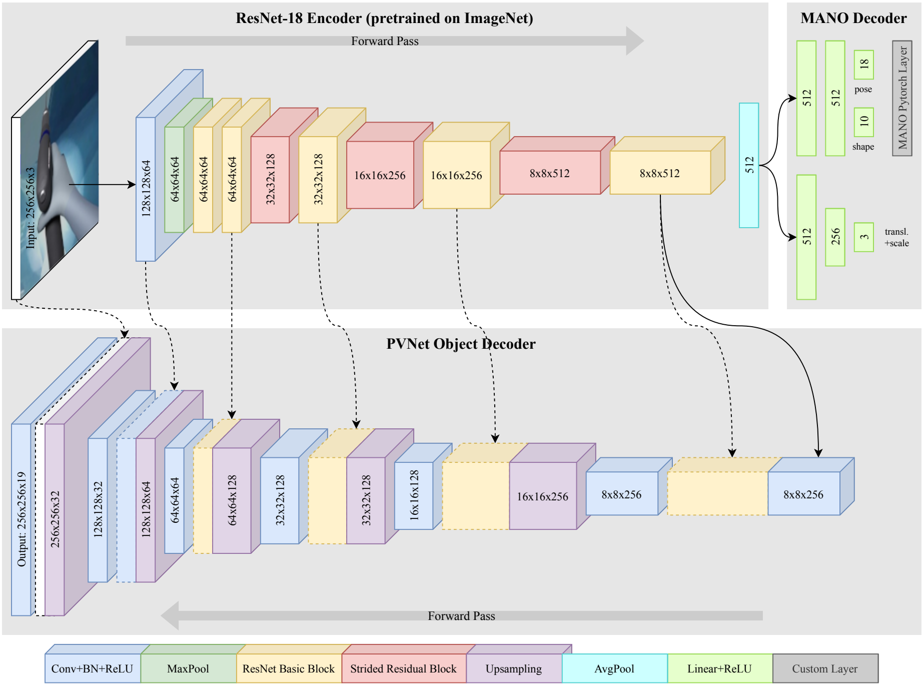

Fig. 1 Overview of the model architecture. The tensor sizes are given as $H \times W \times C$. Skip connections are indicated with dotted lines.

↓ Metric, Model →	HandObjectNet [1]		PVNet [3]		Ours	
	98th-%	Max.	98th-%	Max.	98th-%	Max.
Tool ADD (mm)	28.15	105.86	162.04	1998.93	157.86	3564.66
Tool Proj2D (px)	20.70	1181.93	40.12	3023.87	41.26	7408.28
Drill tip error (mm)	132.61	243.52	455.62	1941.20	460.90	3205.32
Drill bit dir. error (deg)	19.43	40.82	161.17	178.58	167.38	179.53
2D keypoint error (px)	-	-	39.31	191.93	39.55	856.13
Hand ADD (mm)	28.81	113.25	-	-	74.70	143.28
Hand Proj2D (px)	16.56	618.68	-	-	38.20	468.18

Table 1 The 98th percentiles and maximum errors of the models on real test data. All models were trained on synthetic data and refined using real data. The reported percentiles and maxima were aggregated over 5-fold cross validation.

block has a skip connection from the last layer of the shared encoder with the respective feature map size. Hence, the input for the convolutional layer in each block is the concatenation of the encoder feature maps and the upsampled feature maps of the previous convolution in the decoder. While the original PVNet object decoder only downsamples the feature maps to a minimum size of $H/8 \times W/8$, we reverted this design decision to make the decoder branch compatible with the HandObjectNet architecture. The last channel defines a segmentation mask which is used to limit the vector field to the pixels which belong to the tool. For each keypoint we compute the mean and covariance for a set of keypoint candidates, which are generated in a RANSAC-like fashion by repeatedly triangulating two randomly sampled vectors of the vector field. The final 6D tool pose is recov-

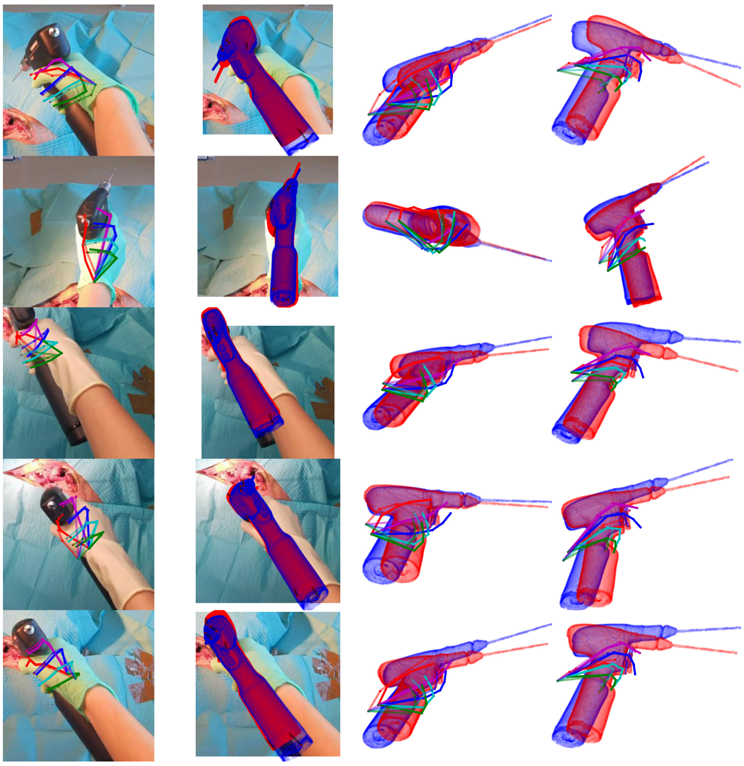

Fig. 2 Qualitative results of HandObjectNet on the real test data. The first column shows the estimated hand pose along with the transparent ground truth. The second column visualizes 2D projections of the ground truth vertices in blue, and the predicted vertices in red. The last two columns display the 3D scene's XZ and YZ subspace and correspond to views from the top and right, respectively. Note that the 3D views are not perspective views, nor was a z-buffer used to compute the correct occlusions.

ered via an uncertainty-driven PnP approach that minimizes the Mahalanobis distance between the estimated distributions and the ground truth 2D keypoints. The RANSAC voting and pose recovery via an uncertainty-driven PnP is adopted from the PVNet paper [3].

We apply the binary cross entropy and vector field losses from the original PVNet paper [3] for the output of the object decoder. For the hand loss we use the mean squared error of the estimated global hand positions, as well as the ℓ_2-regularized MANO pose and shape parameters, as described in [2]. The final loss is composed of the sum of object and hand loss.

The hand joints and mesh representation are computed using the MANO Pytorch layer [2].

2.2 Runtime

To get an estimate of the relative computing efficiency, we measured the inference time for all models and computed the number of trainable parameters. PVNet

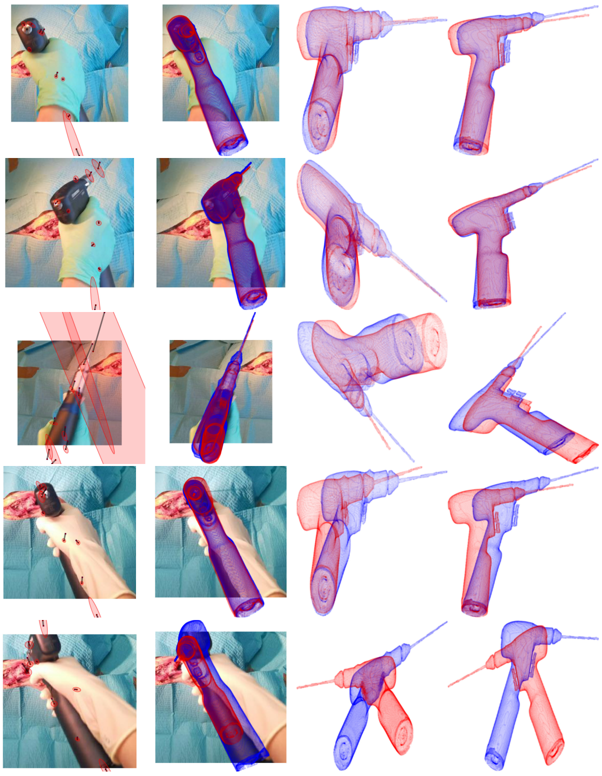

Fig. 3 Qualitative results of PVNet on the real test data. The first column shows estimated 2D keypoints in red, along with their 1σ standard deviation. Each line connects a keypoint estimate to the projection of the corresponding ground truth 3D keypoint. The second column visualizes 2D projections of the ground truth vertices in blue, and the predicted vertices in red. The last two columns display the 3D scene's XZ and YZ subspace and correspond to views from the top and right, respectively. Note that the 3D views are not perspective views, nor was a z-buffer used to compute the correct occlusions.

and HandObjectNet have a comparable size of 13.0×10^6 and 12.5×10^6 trainable parameters, respectively. Our proposed model is slightly larger with 16.4×10^6 trainable parameters, due to additional convolutional layers that were introduced to make the PVNet object decoder branch compatible with the HandObjectNet model. The measured inference time of HandObjectNet is 11.3 fps. PVNet and the proposed model are much slower with 2.6 fps and 2.3 fps, respectively. However, our measurements show that 90 % of the interence time is spent in the RANSAC voting step due to an inefficient, CPU-based implementation of the optimization algorithm. The long runtime is caused by the relatively high number of generated keypoint hypotheses, which are used to reliably estimate the means and covariances for each keypoint. Reducing the number of hypotheses will result in a shorter inference time, at the cost of less accurate uncertainties. All runtime measurements were performed with a batch size of 1 and on a laptop with a Nvidia GTX 1050 Mobile GPU and a Intel i5-8265U CPU.

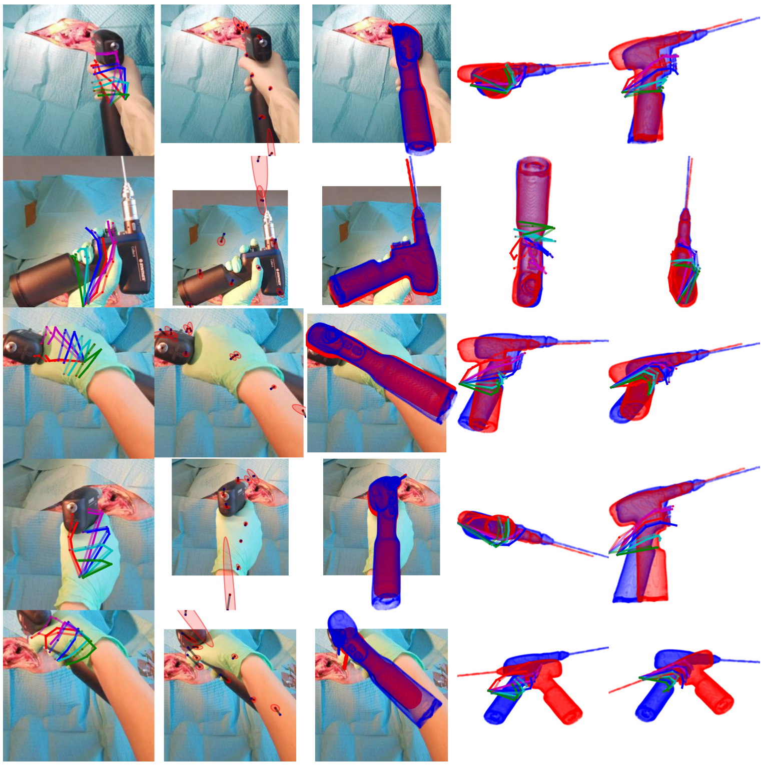

Fig. 4 Qualitative results of our proposed model on the real test data. The first column shows the estimated hand pose along with the transparent ground truth. In the second column, the estimated 2D keypoints are displayed in red, along with their 1σ standard deviation. Each line connects a keypoint estimate to the projection of the corresponding ground truth 3D keypoint. The third column visualizes 2D projections of the ground truth vertices in blue, and the predicted vertices in red. The last two columns display the 3D scene's XZ and YZ subspace and correspond to views from the top and right, respectively. Note that the 3D views are not perspective views, nor was a z-buffer used to compute the correct occlusions.

3 Additional Evaluations

3.1 Qualitative Results

We show exemplary pose estimation results for HandObjectNet, PVNet and the combined baseline, in Figures 2, 3 and 4, respectively. In addition, we visualize the full range of the accuracy-threshold curves for the tool and hand ADD metrics in Figure 5, which are truncated in the main manuscript for easier readability of the plots in the relevant error range for clinical applications. Last, we report the 98th percentiles as well as the observed maximum errors in Table 1 to give an estimate of the worst-case scenario.

We observe two flaws in PVNet's keypoint-based pose estimation. First, the keypoint recovery becomes unstable in case of severe truncation of the tool, which is due to the small area of the vector field. In these cases, the estimated pose can have arbitrarily large errors. Second, the pose recovery via PnP sometimes

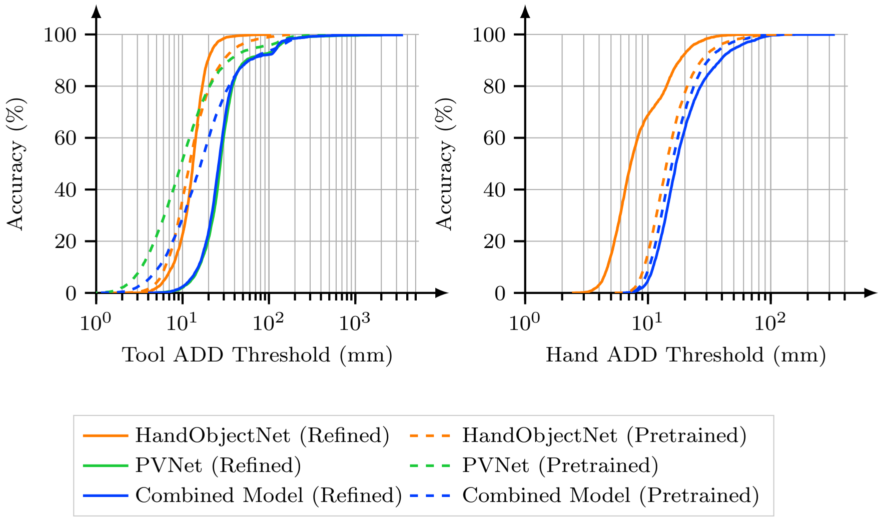

Fig. 5 Accuracy-threshold curves of the tool and hand ADD metrics for all baseline models. Pretrained models are indicated with dashed lines and evaluated on synthetic data. Refined models are indicated with solid lines and evaluated on real data. Please note the log scale on the x axis as well as the extended range compared to the plots in the main manuscript.

	2D keypoint error (px)	Tool ADD (mm)	Tool Proj2D (px)
Pretraining only	37.03 (25.46)	118.64 (65.02)	34.63 (22.97)
100 samples	50.58 (46.51)	186.33 (108.04)	49.47 (33.49)
+ pretraining	35.71 (29.50)	138.60 (45.27)	33.33 (19.21)
1000 samples	23.46 (19.58)	72.00 (27.54)	18.34 (12.74)
+ pretraining	18.96 (15.13)	62.69 (26.03)	22.68 (10.78)
2337 samples	25.01 (20.86)	61.13 (29.22)	32.84 (13.58)
+ pretraining	**18.55** (15.14)	**49.30** (25.10)	**15.56** (10.89)

Table 2 Evaluation of the effects of the real training set size as well as pretraining on synthetic data. We report the average error for our combined model as well as the median error in parentheses.

converges to a pose that is mirrored along the depth axis, as can be seen on the sample in the last row of Figure 3. Due to the similar architecture, we also observe these issues in the estimates of the proposed model. To prevent such flipped pose estimates, a strong prior could be introduced in the uncertainty-PnP.

3.2 Ablation study

To confirm the observations from [5] for our particular scenario, we evaluate the benefit of pretraining with synthetic data in low-data regimes. For this exemplary ablation study, we select the combined model and use a simple train/test split instead of k-fold cross validation. We analyze the effect of the number of real training samples on the model performance as well as the effect of pretraining on synthetic data. The results are summarized in Table 2. Pretraining the model on synthetic data significantly improves the model performance for all tested training set sizes.

Moreover, we found that the pretrained models are less prone to overfitting and more stable during the training process.

References

1. Hasson, Y., Tekin, B., Bogo, F., Laptev, I., Pollefeys, M., Schmid, C.: Leveraging photometric consistency over time for sparsely supervised hand-object reconstruction. In: Proceedings of the IEEE/CVF Conference on Computer Vision and Pattern Recognition (CVPR) (2020)
2. Hasson, Y., Varol, G., Tzionas, D., Kalevatykh, I., Black, M.J., Laptev, I., Schmid, C.: Learning joint reconstruction of hands and manipulated objects. In: Proceedings of the IEEE Conference on Computer Vision and Pattern Recognition, pp. 11807–11816 (2019)
3. Peng, S., Liu, Y., Huang, Q., Zhou, X., Bao, H.: Pvnet: Pixel-wise voting network for 6dof pose estimation. In: Proceedings of the IEEE Conference on Computer Vision and Pattern Recognition, pp. 4561–4570 (2019)
4. Ronneberger, O., Fischer, P., Brox, T.: U-net: Convolutional networks for biomedical image segmentation. In: International Conference on Medical image computing and computer-assisted intervention, pp. 234–241. Springer (2015)
5. Zwingmann, J., Konrad, G., Kotter, E., Südkamp, N.P., Oberst, M.: Computer-navigated iliosacral screw insertion reduces malposition rate and radiation exposure. Clinical Orthopaedics and Related Research **467**(7), 1833 (2009)
